# Type 2 diabetes impairs annulus fibrosus fiber deformation and rotation under disc compression in the University of California Davis type 2 diabetes mellitus (UCD-T2DM) rat model

**DOI:** 10.1093/pnasnexus/pgad363

**Published:** 2023-11-03

**Authors:** James L Rosenberg, Eric Schaible, Alan Bostrom, Ann A Lazar, James L Graham, Kimber L Stanhope, Robert O Ritchie, Tamara N Alliston, Jeffrey C Lotz, Peter J Havel, Claire Acevedo, Aaron J Fields

**Affiliations:** Departments of Mechanical and Biomedical Engineering, University of Utah, Salt Lake City, UT 84112, USA; Advanced Light Source, Lawrence Berkeley Laboratory, Berkeley, CA 94720, USA; Department of Epidemiology and Biostatistics, University of California, San Francisco, CA 94143, USA; Department of Epidemiology and Biostatistics, University of California, San Francisco, CA 94143, USA; Department of Molecular Biosciences, University of California, Davis, CA 95616, USA; Department of Nutrition, University of California, Davis, CA 95616, USA; Department of Molecular Biosciences, University of California, Davis, CA 95616, USA; Department of Nutrition, University of California, Davis, CA 95616, USA; Materials Science Division, Lawrence Berkeley National Laboratory, Berkeley, CA 94720, USA; Department of Materials Science and Engineering, University of California, Berkeley, CA 94720, USA; Department of Orthopaedic Surgery, University of California, San Francisco, CA 94143, USA; Department of Orthopaedic Surgery, University of California, San Francisco, CA 94143, USA; Department of Molecular Biosciences, University of California, Davis, CA 95616, USA; Department of Nutrition, University of California, Davis, CA 95616, USA; Departments of Mechanical and Biomedical Engineering, University of Utah, Salt Lake City, UT 84112, USA; Department of Mechanical and Aerospace Engineering, University of California, San Diego, CA 92093, USA; Department of Orthopaedic Surgery, University of California, San Francisco, CA 94143, USA

**Keywords:** intervertebral disc, collagen, type 2 diabetes, low back pain, small-angle X-ray scattering

## Abstract

Understanding the biomechanical behavior of the intervertebral disc is crucial for studying disease mechanisms and developing tissue engineering strategies for managing disc degeneration. We used synchrotron small-angle X-ray scattering to investigate how changes to collagen behavior contribute to alterations in the disc’s ability to resist compression. Coccygeal motion segments from 6-month-old lean Sprague-Dawley rats ( n=7) and diabetic obese University of California Davis type 2 diabetes mellitus (UCD-T2DM) rats ( n=6, diabetic for 68±7 days) were compressed during simultaneous synchrotron scanning to measure collagen strain at the nanoscale (beamline 7.3.3 of the Advanced Light Source). After compression, the annulus fibrosus was assayed for nonenzymatic cross-links. In discs from lean rats, resistance to compression involved two main energy-dissipation mechanisms at the nanoscale: (1) rotation of the two groups of collagen fibrils forming the annulus fibrosus and (2) straightening (uncrimping) and stretching of the collagen fibrils. In discs from diabetic rats, both mechanisms were significantly impaired. Specifically, diabetes reduced fibril rotation by 31% and reduced collagen fibril strain by 30% (compared to lean discs). The stiffening of collagen fibrils in the discs from diabetic rats was consistent with a 31% higher concentration of nonenzymatic cross-links and with evidence of earlier onset plastic deformations such as fibril sliding and fibril–matrix delamination. These findings suggest that fibril reorientation, stretching, and straightening are key deformation mechanisms that facilitate whole-disc compression, and that type 2 diabetes impairs these efficient and low-energy elastic deformation mechanisms, thereby altering whole-disc behavior and inducing the earlier onset of plastic deformation.

**Significance Statement**

Low back pain is among the leading causes of disability and is often linked to intervertebral disc degeneration. Type 2 diabetes is an independent risk factor for low back pain, disc degeneration, and disc tissue damage, yet the underlying mechanisms remain poorly understood. Here, we show that compressive loading of the whole intervertebral disc is accommodated by nanoscale deformation mechanisms of collagen fibrils, which are compromised by the embrittlement of collagen in type 2 diabetes. These findings provide novel insight into the potential mechanisms underlying diabetes-related disc tissue damage and may inform the development of preventative and therapeutic strategies for this debilitating condition.

## Introduction

Mechanistic understanding of the biomechanical behavior of the intervertebral disc can provide insight into the effects of disc degeneration and inform tissue engineering strategies for repairing or replacing damaged or degenerated discs. The complex biomechanical behavior of the disc reflects its hierarchical structure that spans multiple length scales. Thus, a common experimental strategy for studying disc biomechanical behavior employs a reductionist approach. Specifically, disc substructures are tested ex situ using boundary conditions that mimic the in situ loading environment, and then the experimental stress–strain data are fitted to a multiphase constitutive model that predicts the deformations of the nanoscale tissue constituents (1, 2). However, validating the predicted nanoscale deformations and understanding how those deformations relate to whole-disc biomechanical behavior remain challenging. Here we sought to relate the deformation mechanisms of the nanoscale constituents to whole-disc behavior by simultaneously measuring the nanoscale deformations during whole-disc compression, which is a primary loading mode of spinal discs.

**Fig. 1.**
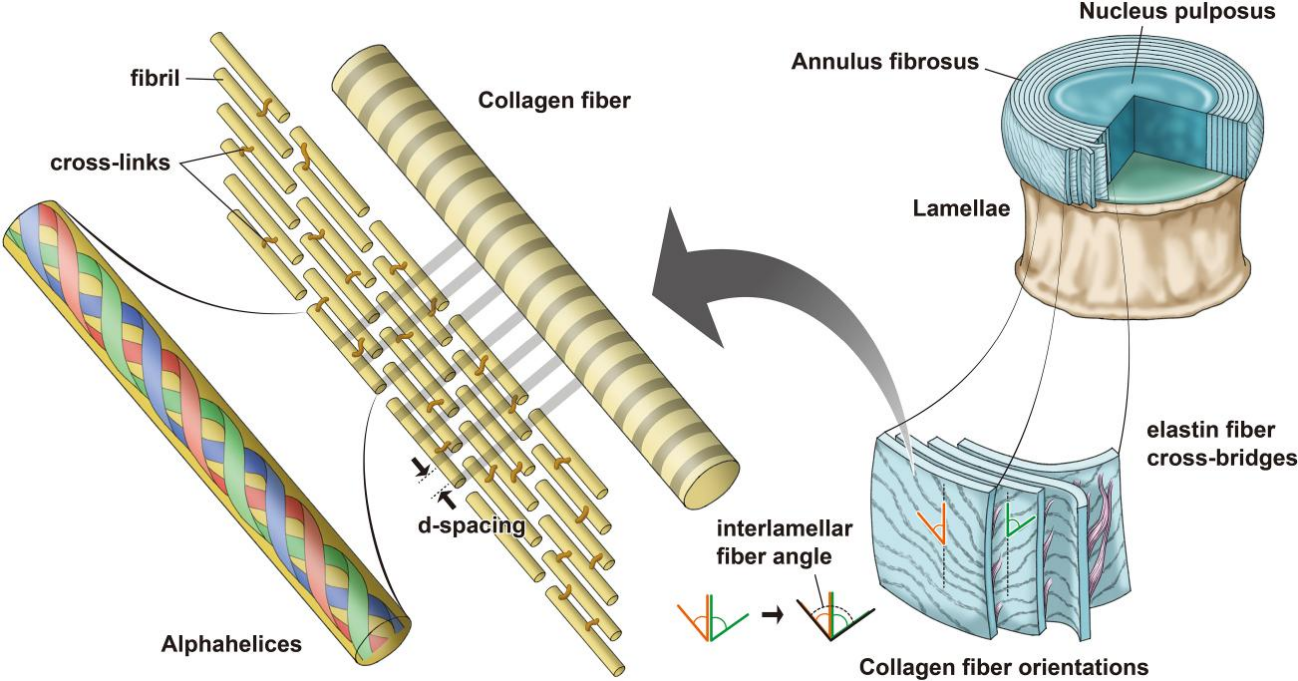
The hierarchical structure of the intervertebral disc (approximately 45–55 mm diameter in the human lumbar spine and 2.5–5 mm in the rat coccygeal spine) is composed of the outer fiber-reinforced annulus fibrosus and inner gelatinous nucleus pulposus core. The type 1 collagen fibers that reinforce the annulus fibrosus have a typical diameter of approximately 1−10μm and are made up of many collagen fibrils with diameters on the order of 100 nm. The fibrils contain collagen alpha-helices, which are each around 1.6 nm in diameter.

**Fig. 2.**
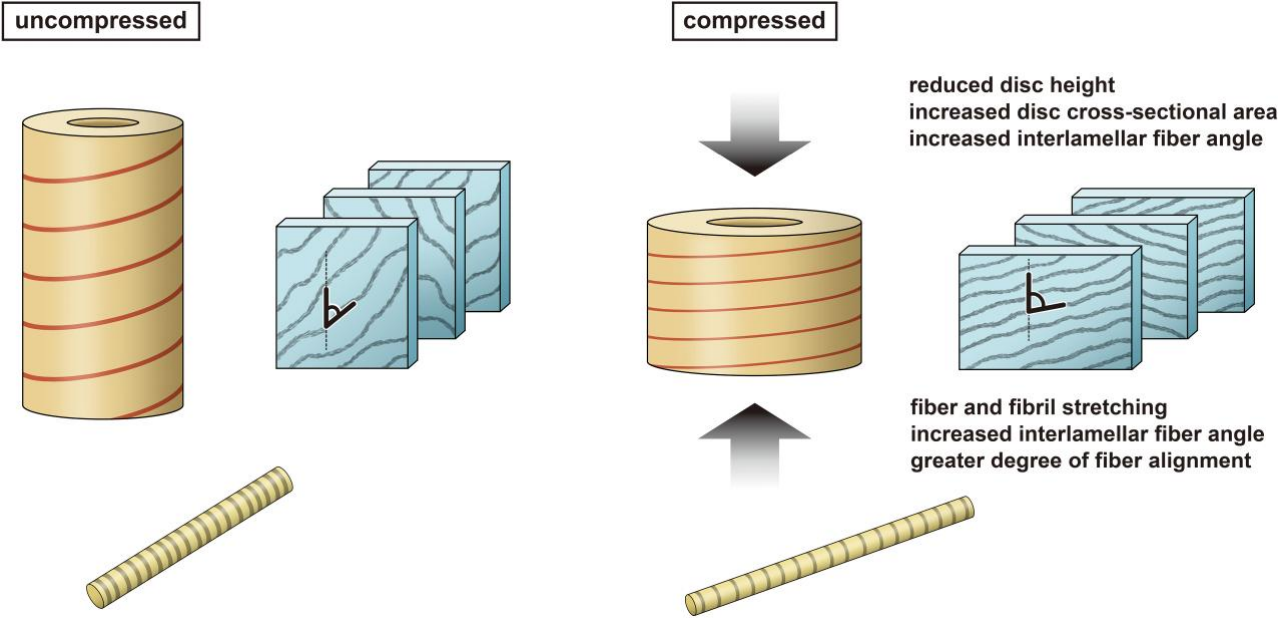
A simplified model of disc compression as a hydrostatic skeleton. Compared to the uncompressed state, compression of the hydrostatic skeleton or disc is theorized to increase intralamellar fiber alignment, increase the interlamellar fiber angle, and increase fibril stretching.

Like many “hydrostatic skeletons” found in nature (3, 4), the intervertebral disc consists of a hydrated inner core, the nucleus pulposus, constrained by outer layers of fiber-reinforced connective tissue, the annulus fibrosus, which control and limit shape change. The collagen fibrils that reinforce the annulus fibrosus are organized in a crossed-fiber helical array in which sheets of collagen fibers wrap the disc in right- and left-handed helices (Fig. 1). Within this crossed-fiber helical organization, the orientation of the collagen fibrils alternates between adjacent annulus fibrosus lamellae at roughly $\pm 60^{\circ }$ angles to the vertical axis (5, 6); fiber angle also changes slightly along the inferior–superior axis of the disc and from the outer layers of the annulus fibrosus to the inner layers of the annulus fibrosis (7). Although the collagen fibers are stiff and relatively in-extensible in tension, the disc can sustain large amounts of compression without fiber rupture or delamination because the pitch of the helix changes (Fig. 2). Specifically, the average angle between fibers of adjacent annulus fibrosus lamellae, or mean interlamellar angle (Fig. 1) increases during disc shortening in compression (8, 9). Klein and Hukins (10) first confirmed the increase in fiber angle by analyzing X-ray diffraction patterns during disc compression.

**Fig. 3.**
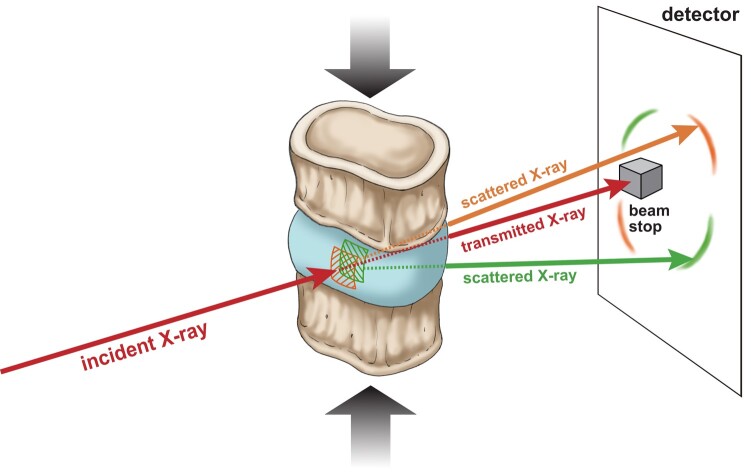
The in situ SAXS compression setup permits simultaneous, real-time measurement of collagen deformation during whole-disc compression. During SAXS acquisition, the high-energy incident X-ray beam is passed through the disc at mid-annulus, and a detector captures the scattering pattern.

Beyond changes in fiber angle, though, little is known about the relative amounts and types of nanoscale tissue deformations that confer energy dissipation during disc compression. In other multiscale soft tissues, e.g. skin, nanoscale deformations such as fibril straightening, alignment, sliding, and stretching play important roles, with fibril straightening and alignment predominating at lower applied strains, and fibril sliding and stretching predominating at higher strains (11). In the disc, a prior study inferred modest sliding of collagen fibrils on the surface of the annulus fibrosus during applied flexion (12, 13). However, because it was difficult to resolve individual collagen fibrils, the relative amounts of any straightening, alignment, and stretching remain unclear. In the present study, we addressed this issue using synchrotron small-angle X-ray scattering (SAXS). This approach permits simultaneous, real-time measurement during the compression test of the deformations and strains in the collagen fibrils as compared to whole-disc compression (Fig. 3). Using this approach, this study sought to (1) provide a quantitative description of collagen fibril deformation during modest amounts of whole-disc compression and (2) determine how alterations in collagen fibril cross-linking caused by type 2 diabetes mellitus (T2DM) impair fibril deformation. T2DM is a global epidemic that increases cross-linking in many collagenous tissues, including the disc (14, 15).

## Results

### Diabetes alters whole-disc biomechanical behavior

**Fig. 4.**
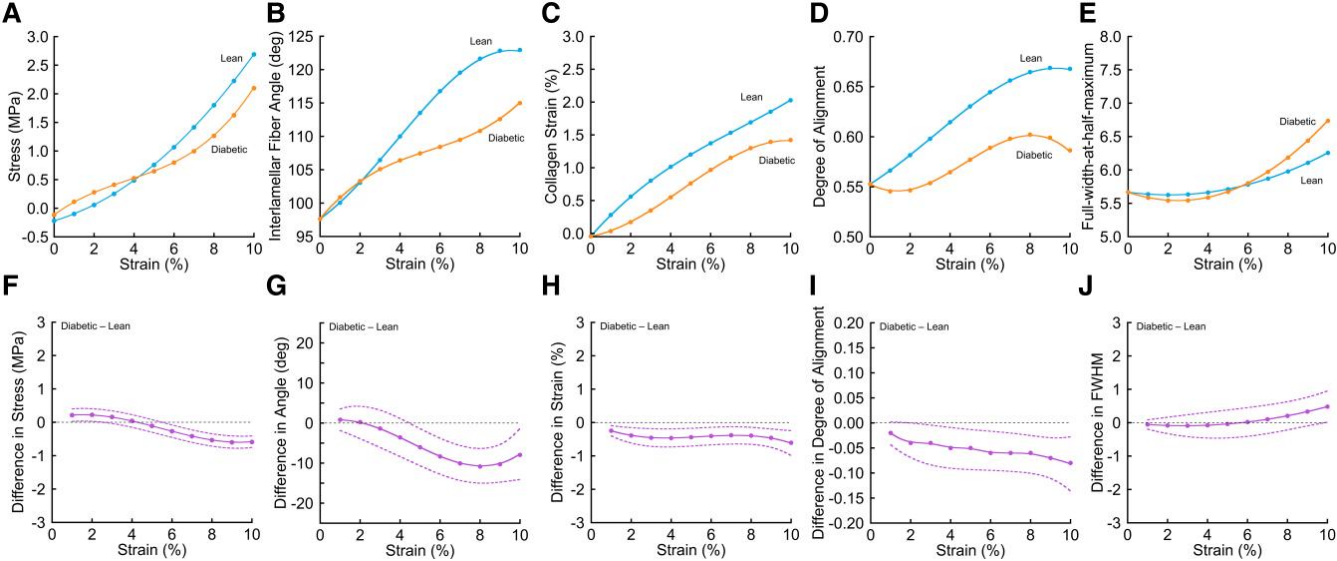
Comparison of whole-disc and nanoscale biomechanical behavior between discs from lean vs. diabetic rats. Mean stress (A), interlamellar fiber angle (B), collagen strain calculated from the measured *d*-spacing of the gap plus overlap in collagen fibrils (C), degree of alignment (D), and full-width-at-half-maximum (E) as a function of applied compressive strain. Data points show the mean value for discs from lean rats ( n=7 rats) and diabetic rats ( n=6 rats). (F)–(J) Difference in mean value (solid curves, with 95% confidence intervals, dashed bounds) as a function of applied compressive strain. Diabetic minus lean; >0 indicates higher mean value in the discs from diabetic rats. Dashed horizontal line indicates no difference. Compared to lean rats, discs from diabetic rats had significantly altered stress response (F), reduced mean interlamellar angle (G), less collagen fibril strain (H), reduced fibril alignment (I), and greater distributions of *d*-spacing at higher applied strains, suggestive of fibrillar sliding and delamination (J).

Discs from lean rats exhibited the nonlinear stress–strain behavior that is seen in other collagenous tissues (11, 16–18), with the three characteristic toe, heel, and linear regions (Fig. 4A). By comparison, discs from the diabetic rats exhibited a stress response without the flat toe region and pronounced heel region (Fig. 4A and F). Specifically, in the toe stage between 0 and 2% applied strain, the discs from the diabetic rats had significantly higher axial compression stress. In the heel stage between 2 and 5% applied strain, the discs from the diabetic rats continued to have higher axial compressive stress, but the difference between groups lessened as the discs from the lean rats developed increasing stiffness. In the linear stage between 5 and 10% applied strain, discs from both rat strains had roughly linear stress–strain curves, the axial stress being significantly higher for the discs from the lean rats. In the linear stage, there was a slight but noticeable increase in the slope of the stress–strain curve for the discs from diabetic rats, and nearing 10% applied strain, the slope of the stress–strain curve appeared to exceed that of the discs from lean rats.

**Fig. 5.**
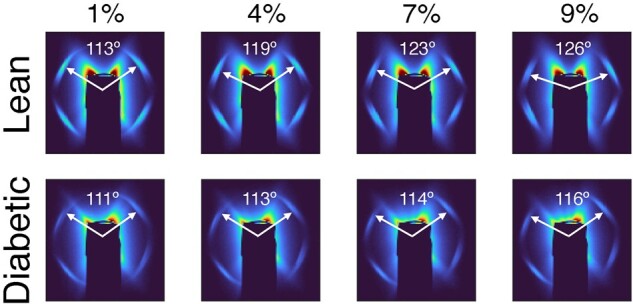
Comparison of X-ray scattering patterns between a representative disc from each group showing the relative peak positions at various amounts of disc compression. Applied compressive strains and interlamellar fiber angles are shown.

In order to determine how the collagen fibrils responded to increasing amounts of compressive strain, we analyzed the evolving multipeak scattering patterns that were collected during loading (Fig. 5). By measuring the changing radial positions, azimuthal angles, and azimuthal intensity distributions of the peaks, we made direct measurements of the ways in which the fibrils stretched, rotated, and realigned in response to the increasing compressive strain at the whole-disc level.

### Diabetes significantly decreases the mean interlamellar angle in the heel and linear stages

The interlamellar fiber angle was significantly different between discs from diabetic vs. lean rats (Fig. 4B and G). Specifically, the interlamellar fiber angle in the unloaded discs was similar in both groups (approximately $97^{\circ }$) but diverged as the strain was applied. Applied compressive strains of up to approximately 3% were accommodated by a $10^{\circ }$ increase in the interlamellar angle in both groups, indicative of a similar increase in fiber angle relative to the vertical axis (Fig. 4B). Beyond 3% applied strain, mean interlamellar fiber angle continued to increase in the discs from the lean rats. However, in the discs from the diabetic rats, the increase in mean interlamellar fiber angle was significantly more gradual, as the additional applied strain was accommodated by smaller increases in interlamellar angle. By 10% applied strain, the overall increase in interlamellar fiber angle compared to the unloaded state was 31% less in the discs from diabetic rats ( $18^{\circ }\pm 3.1^{\circ }$ vs. $26^{\circ }\pm 2.5^{\circ },P=0.011$; Figs. 4G and 5).

### Diabetes significantly reduces collagen fibril stretching and leads to earlier plastic responses

We also used the SAXS data to measure collagen fibril strain, calculated from the change in *d*-spacing, in comparison to the applied macroscopic compressive strain. In both groups of discs, collagen fibril strain increased approximately linearly with applied compressive strain (Fig. 4C), but applied compression was accommodated by significantly more collagen stretching in the discs from the lean rats (Fig. 4H). By 10% applied compressive strain, collagen fibril stretching was 30% lower in the discs from the diabetic rats ( 1.4±0.17% vs. 2±0.11%,P=0.001). In other words, 20% of the applied strain was transferred to the collagen fibrils in the lean group vs. only 14% in the diabetic group. The reduced ability of the diabetic discs to transfer tissue strain into nanoscale fibril strain was accompanied by evidence of earlier transition to plastic responses associated with damage. By measuring the width of the SAXS peaks (full-width-at-half-maximum, FWHM), we measured the difference in the distribution of *d*-spacings within the discs. In diabetic discs, this distribution broadened at strains greater than 4% (Fig. 4E), suggesting that interfibrillar and intrafibrillar sliding and fibril delamination were occurring, and group differences in FWHM reached statistical significance at 10% applied strain (Fig. 4J). This evidence of an earlier transition to plastic (nonrecoverable) responses was further supported by Fig. 4C, which shows the collagen fibrils of the diabetic discs transitioning from a linear stretching regime to a plateau at strains above 8%, and by Figs. 4D and 4I, which show the fibrils in the diabetic discs falling out of common alignment at strains above 8%.

### Diabetes significantly increases nonenzymatic cross-links in the disc

In discs from the diabetic rats, the smaller amount of fiber rotation (i.e. the smaller changes in mean interlamellar fiber angle), greater fibril stiffness, and reduced straightening coincided with a higher amount of collagen cross-linking. Specifically, discs from diabetic rats had 31% higher concentration of nonenzymatic cross-links, or advanced glycation end products (AGEs), in the annulus compared to discs from lean controls ( 237±25ng/mg vs. 310±17ng/mg,P=0.007). This is consistent with our previous findings linking hyperglycemia to accelerated AGE accumulation in the disc (14).

## Discussion

Here we measured how disc compression at the mesoscale is accommodated by deformations of the collagen fibrils at the nanoscale. Using high-energy synchrotron X-ray scattering measurements during monotonic disc compression, we observed distinct collagen deformations that coincided with the toe, heel, and linear stages of whole-disc stress–strain behavior. In discs from healthy rats, these deformations included increases in fiber tilt (i.e. increases in the mean interlamellar angle) and fibril straightening and stretching. Whereas a previous study observed that 10–15% disc compression is accommodated by increases in annulus fibrosus fiber tilt (10), our new data show that similar amounts of whole-disc compression simultaneously straighten (uncrimp) and stretch the collagen fibrils in the annulus fibrosus too, which suggests those are also important nanoscale mechanisms of energy absorption during moderate disc compression. Our findings also demonstrate that the nanoscale deformations are altered by diabetes. Specifically, discs from rats with type 2 diabetes showed a 31% lower increase in mean interlamellar fiber angle, 30% less collagen fibril stretching, and less fibril straightening and uncrimping. Taken together, these findings show that fibril reorientation, stretching, and straightening are key deformation mechanisms that facilitate whole-disc compression and that type 2 diabetes impairs these efficient and low-energy elastic deformation mechanisms, thereby altering whole-disc behavior and inducing the earlier onset of plastic deformation.

A novel finding of this study is that diabetes significantly altered collagen fibril deformation. In the discs from healthy rats, increases in the mean interlamellar angle, collagen fibril stretching, and fibril straightening coincided with a distinct toe region in the stress–strain curve at smaller applied strains, suggesting that these energy-dissipation mechanisms contribute to the disc’s ability to accommodate small amounts of compression with minimal resistance. These nanoscale deformation mechanisms are also important for dissipating energy in skin (11) and tendon (19). Diabetes reduced collagen stretching and straightening (uncrimping), and along with a less hydrated and stiffer nucleus pulposus (14), this led to increased resistance to compression at smaller applied strains (0–2%). At larger applied strains (6–8%), there was evidence of plastic damage onset in diabetic discs. Plastic damage may reflect the collagen fibrils sliding past each other and/or delamination between the collagen and surrounding matrix. This was observed as a plateau in the degree of alignment (Fig. 4D), the increasing dispersion of collagen strains, i.e. increased FWHM (Fig. 4E), and increased stiffness of the collagen (Fig. 4C).

In the discs from diabetic rats, the different collagen deformation mechanisms also coincided with a 31% higher concentration of AGEs, on average. Results from previous studies indicate that diabetes (14) and AGE ingestion (20, 21) accelerate AGE accumulation in the disc, increase disc stiffness, and cause widespread matrix damage (20). In addition, AGEs may cause fibril disruption and collagen degradation (22). Our current results extend those prior findings by showing that greater AGE levels in the disc are associated with increased collagen fibril stiffness, impairments in collagen straightening, and increases in collagen sliding, and that these deficits coincide with alterations to whole-disc compressive behavior.

Another notable finding was that diabetes was associated with significantly less change in the mean interlamellar angle. The nucleus pulposus is highly hydrated. Compressive loading over short time periods produces little fluid flow, so the incompressible fluid induces lateral bulging. Analytical models and experimental measurements on whole discs (10, 23) suggest that this shape change is accommodated by an increase in annulus fibrosus collagen fiber tilt. In agreement with those prior studies, for small-to-moderate reductions in disc height (up to 6–7%), we found large increases in the interlamellar fiber angle in the discs of healthy rats. However, in discs from diabetic rats, the angular changes were much smaller—just $1.8^{\circ }$ for every percent applied strain, compared to $2.6^{\circ }$ for every percent applied strain in the discs from the healthy rats. One possible explanation for the reduced change in mean interlamellar angle is an alteration to the structure or integrity of the interlamellar elastin cross-bridge network, which plays an important role in limiting interlamellar sliding (24). For example, increased cross-linking of the elastin network (25) with diabetes could theoretically impair interlamellar sliding. Based on these collective findings, disc tissue engineering strategies should strive to minimize the resistance to interlamellar sliding as this is an efficient and low-energy deformation mechanism that permits large amounts of disc compression.

This study had several limitations. First, owing to the operational limits of the axial load cell, the maximum applied strain was limited to 10 %, and the mechanisms of collagen deformation and the effects of diabetes could be more pronounced at larger applied strains. Related, we focused on monotonic loading since synchrotron radiation exposure during the testing prevented repeated or cyclic loading of the discs. Cyclic loading could accentuate differences in mechanical behavior that relate to nanoscale damage formation and accumulation. We also focused on the effects of applied compression, and the relative amounts and types of nanoscale deformation mechanisms that occur during applied flexion and torsion are also clinically relevant and remain unclear.

An important technical limitation is that we assumed homogeneous deformations through the annulus fibrosus because the SAXS peak is an integrated measure of X-ray beam scattering generated by all of the lamellae. This approach ignores local differences in collagen alignment and tensile properties of the lamellae, which could vary radially. For example, the outer lamellae are up to three times stiffer than the inner lamellae (7, 26), and the anterior lamellae are stiffer than the posterolateral lamellae (27). These stiffness gradients are believed to reflect regional variation in the type and quantity of collagen fibrils, as type I collagen fibrils predominate in the outer lamellae, whereas smaller type II collagen fibrils, proteoglycans, and water make up the inner lamellae (25, 28). This variation in annulus fibrosus composition may contribute to the within-disc heterogeneity in collagen deformations observed at a single applied compressive strain. In addition to radial heterogeneity, there is also heterogeneity in the inferior–superior direction; for example, the angle and behavior of the fibers at the disc mid-plane may differ from that at the disc-endplate junction (6, 7). To help minimize the confounding effects of this inferior–superior heterogeneity on our SAXS measurements, all of the discs were situated so the beam passed through the disc mid-plane. Related, disc compression involves load sharing between the annulus fibrosus and nucleus pulposus, and thus, some of the differences in annular collagen behavior may reflect concomitant stiffening of the nucleus pulposus due to changes in its fluid-like behavior.

The analysis of degree of alignment (Fig. 4D) reflects changes resulting from both fiber rotation and uncrimping, and this is an additional limitation because it is not possible to distinguish between their respective contributions. Nevertheless, we believe that uncrimping had a relatively minor effect on the observed changes. In skin, for example, where collagen fibrils exhibit extensive crimping, there is a complete ring of intensity in X-ray scattering (11). The absence of a complete ring of intensity in the present study suggests that crimping was either relatively scarce or that it was constrained to smaller angular deviations in fibrils that remained aligned in the two orientations present in the annulus fibrosus. Despite the challenges of disentangling the contributions of fiber rotation from crimping, our analysis provides a reasonable estimate of the extent to which the fibrils are aligned.

In conclusion, our findings demonstrate how compressive loading of the intervertebral disc is accommodated by a variety of nanoscale deformation mechanisms, and our findings quantify how those deformation mechanisms are impaired by type 2 diabetes. Overall, these observed phenomena imply that type 2 diabetes acts to embrittle the collagen and diminish its ductility. Importantly, the hierarchical, multiscale structure of the disc dictates—and our measurements show—that this embrittlement plays a consequential role in how the whole disc resists compression. In particular, we found that type 2 diabetes altered two important collagen deformation mechanisms: (1) diabetes reduced the amount of rotation (i.e. tilt) of the two groups of collagen fibrils that form the annulus fibrosus and (2) diabetes stiffened the collagen fibrils, i.e. less collagen deformation was observed for a given applied tissue strain. Owing to these alterations, there is less energy dissipation via fibril re-alignment, stretching, and rotation, and consequently, we observed earlier onset of plastic deformations such as fibril sliding and fibril–matrix delamination, and we observed a whole-disc stress–strain response that lacked a flat “toe” region. These biomechanical differences coincided with increased AGE content. While it is generally known that type 2 diabetes increases AGE content and collagen stiffness, our findings suggest for the first time how these changes may lead to collagen damage and to alterations in whole-disc compressive behavior.

## Materials and methods

### Animals and samples

Animals were maintained and studied in accordance with Institutional Animal Care and Use Committee-approved protocols at the University of California, Davis. Coccygeal motion segments (CC7–CC8) were harvested from 6-month-old lean Sprague-Dawley rats (LSD; “lean control;” n=7 rats) and diabetic obese UCD-T2DM rats (“diabetic;” diabetic for 68±7 days; n=6 rats) after euthanasia with pentobarbital (200 mg/kg). Of the 13 rats used in this study, 9 rats ( n=4 lean; n=5 diabetic) were used in a prior study of diabetic bone quality (29). The UCD-T2DM rat is a well-validated model of polygenic obese type 2 diabetes that was developed by crossing nondiabetic obese Sprague-Dawley rats with nondiabetic Zucker Diabetic Fatty-lean rats and selectively breeding the offspring to enrich for diabetes (30). The subsequent generations of UCD-T2DM rats demonstrate diabetes in both sexes with adult-onset obesity, insulin resistance, hyperglycemia, and eventual beta cell decompensation. We previously reported that coccygeal discs from different 6-month-old UCD-T2DM rats with a similar duration of diabetes (69±7 days) had reduced glycosaminoglycan content, lower tissue hydration, compromised matrix homeostasis, and diminished creep behavior compared to discs from LSD controls (14).

### Synchrotron small-angle X-ray scattering

Isolated motion segments composed of a single disc surrounded by two vertebrae were used for compression testing. The discs were speckle-coated with India ink, and the two surrounding vertebrae were embedded in blocks of casting polymer, so as to enable the vertebra to be securely clamped into the jaws of a TST350 tensile testing stage (Linkam Scientific). Discs were then loaded in uniaxial compression at a rate of 0.25% strain/second while performing synchrotron X-ray scattering measurements at beamline 7.3.3 of the Advanced Light Source (11). During testing, the midsection of the ventral side of the annulus was intermittently exposed to a 10 keV X-ray beam ( 850×300μm) for 0.5 s at 3-s intervals. The beam passed through the mid-height of the disc and covered roughly 20 % of the height of the discs, on average. The resulting scattering patterns were collected using a Pilatus X-ray detector (models 2M and 1M, Dectris Ltd). Load and displacement data were gathered from the tensile testing stage, and visible light images of the disc were collected using a CCD detector (Allied Vision).

### In situ SAXS data analysis

The X-ray scattering data enabled the measurement of the nanoscale deformations of the collagen under load, including collagen reorientation, alignment, rotation, and stretching. Analyses were performed using the Nika macros for Igor Pro (31) for image calibration, and custom software written in LabVIEW (National Instruments) for subsequent measurements. The X-ray scattering patterns showed characteristic arcs in two primary orientations, corresponding to the left- and right-handed collagen helices present in the annulus fibrosus lamellae. The angles at which the arcs were oriented, and the interlamellar angle between them, were found by fitting the azimuthal intensities of the arcs with Gaussian functions. The mean interlamellar angle was calculated from the difference in angles between the peaks of the curve-fit of the two arcs. This relative measure helped to compensate for any tilt in the sample during loading, which could otherwise confound absolute angle measurements between the annulus fibrosus fibers and the vertical axis. Also, since there is a range of angles at which the fibrils can be oriented within the tissue, this approach captures the predominant interlamellar fiber angle while acknowledging the variability in their distribution. The strain in the collagen was found by measuring the radial distance of the arcs from the beam center, which changes as the *d*-spacing (regular molecular spacing, Fig. 1) of the collagen fibrils lengthens under tension. The arcs were radially integrated into $10^{\circ }$-wide sectors, and the resulting curves were fitted with exponentially modified Gaussian functions. Since the images were calibrated, the radial distance was expressed as a *q*-value, and the *d*-spacing or *d*-period of the collagen fibrils could be calculated as *d*-period = 2pi/*q*. This *d*-spacing includes both the hole and overlap zone. The fibril strain was then calculated as the change in the *d*-period divided by the original *d*-period. The scattering of the *d*-periods was calculated using the FWHM of the collagen peak, which becomes broader as a wider range of *d*-periods appears. The amount of fibril alignment was quantified using an algorithm that sorted the values of the azimuthal intensities, measured the extent to which the total azimuthal intensity fell into fewer bins, and normalized the resulting values onto a scale from 1 (all intensity in one bin) to 0 (intensity spread evenly across all bins).

### Whole-disc behavior

Load–displacement data collected during SAXS were converted to whole-disc stress–strain curves. Stress was calculated from the disc cross-sectional area using pretest radiographs. We tested discs that had an average diameter of 3.5±0.7mm and an average height of 1.4±0.4mm. The axial compressive strain was calculated from high-resolution images of the speckle pattern captured during the loading. The compressive tissue strain was determined from the images of the speckle pattern using a modified 1D digital image correlation algorithm in Matlab (Mathworks).

### Biochemical analysis of cross-links

Advanced glycation end products (AGEs) arise as a result of nonenzymatic glycation of the free amino groups of proteins and lipids by reducing sugars and acting as intermolecular cross-links. A fluorimetric assay was used to measure the total concentration of AGEs in bulk samples of the annulus fibrosis (lean: n=6 rats, diabetic: n=4 rats). Excised annulus samples were first hydrolyzed in 6N HCl (24 h, $110^{\circ }C$). Fluorescence readings of the neutralized lysates were taken using a SpectraMax M5 spectrophotometer (Molecular Devices, Sunnyvale, CA) at the excitation wavelength of 370 nm and emission wavelength of 440 nm (32–34). These fluorescence readings were referenced to a quinine sulfate standard and then normalized to the collagen content in the same lysates. Collagen content was calculated from the amount of hydroxyproline, which we determined using a chloramine T colorimetric assay (34).

### Statistics

Whole-disc biomechanical outcomes (axial compressive stress) and nanoscale biomechanical outcomes (interlamellar fiber angle, collagen strain, degree of alignment, FWHM) were compared between discs from lean control and diabetic UCD-T2DM rats using mixed models. A separate regression model for each outcome (vs. applied strain) with random intercepts was fit. We flexibly modeled trajectories by testing whether including quadratic or cubic terms for strain improved the model fit and included them if indicated by a significant ( P<0.05) likelihood ratio test. Kenward–Roger denominator degrees of freedom were used. We estimated whether trajectories differed by study group (lean vs. diabetic) using main effects, study group and strain, and interaction between the study group and strain in the model. We assessed whether differences in trajectories between groups occurred using an F-test via the SAS contrast statement to simultaneously test the differences in the slopes and intercepts (35). We assessed whether the log transformation of each outcome or centering strain improved the model fit, and we found that the nontransformed and noncentered models provided similar results, and therefore for ease of presentation, the simpler models are presented. Estimates of the group mean and between-group differences and 95% confidence intervals are generated from these models. A.A.L. and A.B. designed the statistical analyses and A.B. performed the mixed model analyses in SAS v. 9.4 proc mixed. Significance was defined by P<0.05 (2-tailed). All data are given as mean ± SD.

## Data Availability

The data that support the findings in this publication are publicly available on the open-source data repository Dryad.
